# Bone Morphogenetic Protein Signaling in Cancer; Some Topics in the Recent 10 Years

**DOI:** 10.3389/fcell.2022.883523

**Published:** 2022-05-25

**Authors:** Shogo Ehata, Kohei Miyazono

**Affiliations:** ^1^ Department of Pathology, School of Medicine, Wakayama Medical University, Wakayama, Japan; ^2^ Department of Applied Pathology, Graduate School of Medicine, The University of Tokyo, Tokyo, Japan

**Keywords:** BMPs, ALKs, Smads, cancer, metastasis, angiogeneis

## Abstract

Bone morphogenetic proteins (BMPs), members of the transforming growth factor-β (TGF-β) family, are multifunctional cytokines. BMPs have a broad range of functions, and abnormalities in BMP signaling pathways are involved in cancer progression. BMPs activate the proliferation of certain cancer cells. Malignant phenotypes of cancer cells, such as increased motility, invasiveness, and stemness, are enhanced by BMPs. Simultaneously, BMPs act on various cellular components and regulate angiogenesis in the tumor microenvironment. Thus, BMPs function as pro-tumorigenic factors in various types of cancer. However, similar to TGF-β, which shows both positive and negative effects on tumorigenesis, BMPs also act as tumor suppressors in other types of cancers. In this article, we review important findings published in the recent decade and summarize the pro-oncogenic functions of BMPs and their underlying mechanisms. The current status of BMP-targeted therapies for cancers is also discussed.

## Introduction

Bone morphogenetic proteins (BMPs) were originally identified as bone- and cartilage-inducing factors in the bone matrix (Wozney et al., 1988; reviewed in; Katagiri & Watabe, 2016). Subsequent studies revealed that BMPs exert a wide range of biological effects. Since then, a variety of BMP roles have been shown in cancer progression. BMPs induce the proliferation of several types of cancer cells, suggesting that BMPs act as pro-tumorigenic factors. BMPs enhance malignant phenotypes of cancer cells, such as cell motility and invasiveness. BMPs also act on various cellular components in the tumor microenvironment, regulating angiogenesis and the immune landscape. In contrast, BMPs serve as tumor suppressors in certain types of cancers. These divergent roles of BMPs have been discussed in many other review articles, including our previous review articles (Ehata, et al., 2013; Davis et al., 2016). However, the precise mechanisms for the pro-oncogenic or tumor-suppressive functions of BMPs remain to be elucidated.

## Bone Morphogenetic Protein Signaling Pathway

**FIGURE 1:**
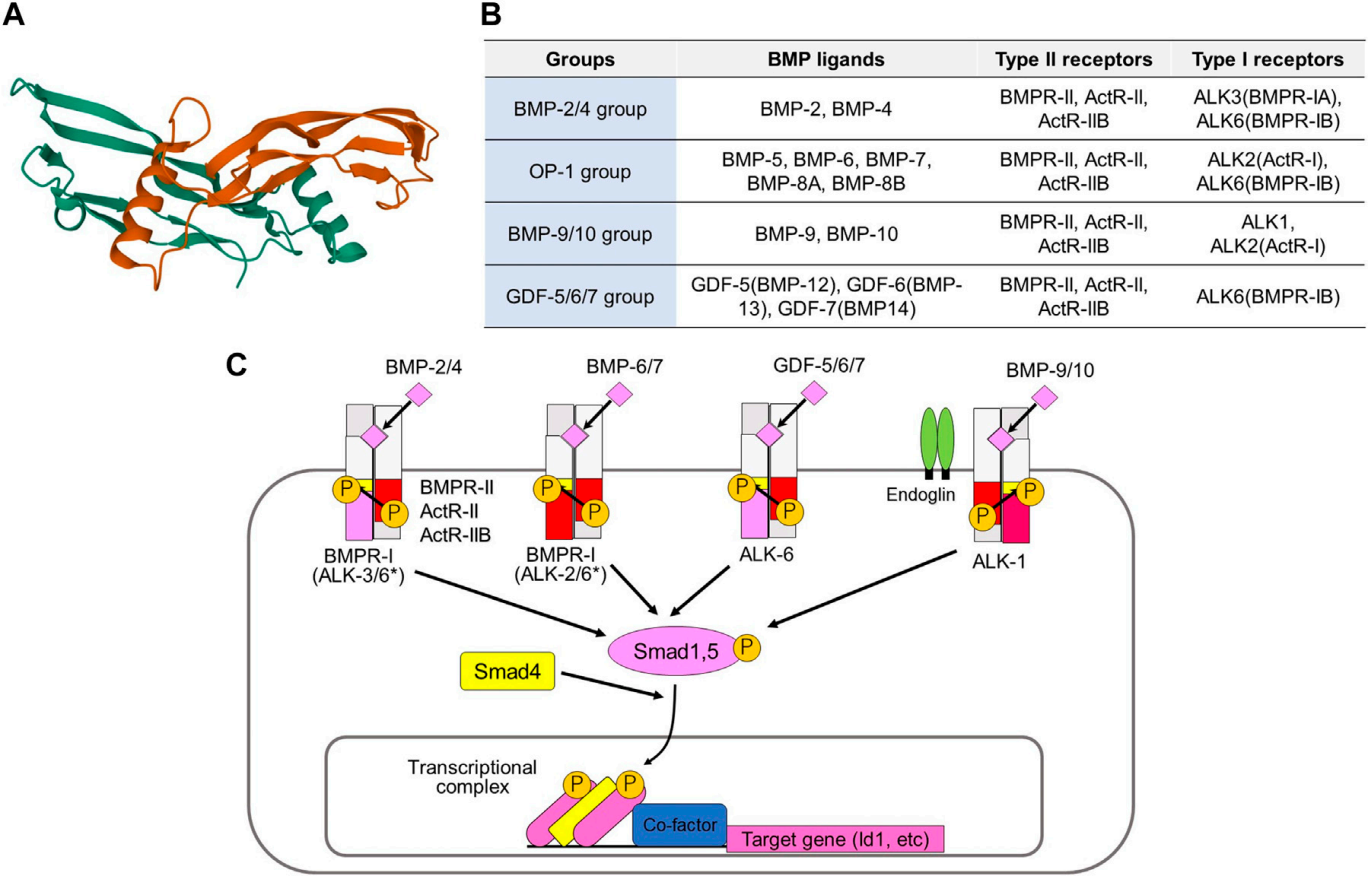
Activation of BMP signaling pathways by various BMP ligands and type II and type I receptors. **(A)** Three-dimensional structure of human BMP-6 homodimer. This figure is created based on the PDB information deposited by Juo and Seeherman (https://www.rcsb.org/structure/6OMO). **(B)** Classification of BMPs based on their binding affinities to receptors. **(C)** BMPR-II, ActR-II, and ActR-IIB are type II receptors, while ALK1, ALK2, ALK3, and ALK6 are type I receptors for BMPs. The binding profiles of BMP-2/4 group, the BMP-5/6/7/8 group, the GDF-5/6/7 group, and the BMP-9/10 group to type I receptors are shown. *BMPs of BMP-2/4 group preferentially bind to ALK3 and ALK6, while those of OP-1 group mainly bind to ALK2 and ALK6. However, BMP-2/4 may transduce signals through ALK2, while OP-1 group ligands may transduce signals through ALK3 under certain conditions. Type II and type I receptors form a heteromeric complex and transduce intracellular signals by phosphorylating Smad1/5, as well as through non-Smad signaling pathways. The phosphorylated Smads form complexes with Smad4 and associate with various transcription factors and transcriptional coregulators in the nucleus, thereby regulating transcription of the target genes, including ID1.

BMPs, which are members of the transforming growth factor-β (TGF-β) family, are multifunctional cytokines. More than a dozen BMPs have been identified in vertebrates (Figures 1A,B) (Miyazono et al., 2010; Morikawa et al., 2016). Several BMPs are also known as osteogenic proteins (OPs) or growth differentiation factors (GDFs). BMPs bind to two different groups of cognitive kinase receptors, namely type I and type II TGF-β family receptors, both of which are required for signal transduction. Unlike TGF-β, certain BMPs can bind to type I receptors in the absence of type II receptors. However, the binding affinities of BMPs to type I receptors are facilitated by the presence of type II receptors. Among the five different type II receptors in mammals, BMPs bind to BMP type II receptor (BMPR-II), activin type II receptor (ActR-II), and activin type IIB receptor (ActR-IIB). Among the seven type I receptors, BMPs bind to activin receptor-like kinase (ALK) 1, 2, 3, and 6 (Figures 1B,C).

BMPs can be classified into four subgroups according to their structural similarities and ability to bind to type I receptors (Miyazono et al., 2010; Sanchez-Duffhues et al., 2020). The BMP-2/4 group preferentially binds to ALK3 and ALK6, whereas the BMP-5/6/7/8 group mainly binds to ALK2 and ALK6. The GDF-5/6/7 (also known as BMP-12/13/14) group binds to ALK6, but does not bind to other type I receptors. The BMP-9/10 group binds to ALK1 and binds weakly to ALK2. BMP signaling is modified by membrane proteins, as well as by diverse secreted proteins (Brazil et al., 2015; Correns et al., 2021). A co-receptor, endoglin (also known as CD105) upregulates BMP-9/10-ALK1 signaling in endothelial cells and downregulates TGF-β-ALK5 signaling (Dallas et al., 2008). BMP and activin membrane bound inhibitor (BAMBI) acts as a pseudoreceptor and negatively regulates BMP signaling. Structurally diverse secreted proteins, such as noggin, chordin, and gremlin1, bind directly to BMPs and regulate their availability as antagonists (Chang et al., 2016).

Upon binding to type I and type II receptors, BMPs form heterotetrameric receptor complexes (Miyazono et al., 2010). Type II receptor serine/threonine kinases are constitutively active, and they activate type I receptor kinases through phosphorylation of the Gly-Ser-rich (GS) domain of type I receptors. Type I receptors activate receptor-regulated Smads (R-Smads: Smad1 and Smad5). Although Smad8 (also known as Smad9) is structurally similar to Smad1 and Smad5, its function has not been fully elucidated. Recent findings have suggested that Smad8 acts as an antagonist of Smad1/5 (Tsukamoto et al., 2014). Some mutations affect residues in the GS domain or the ATP-binding pocket of the kinase domain of ALK2, leading to increased phosphorylation of R-Smads (Wu et al., 2014; Sanchez-Duffhues et al., 2020). Phosphorylated R-Smads induce heteromeric assembly with the common partner Smad (Co-Smad; Smad4). R-Smad/Co-Smad complexes bind to various transcription factors and transcriptional co-activators [p300, cAMP response element-binding protein-binding protein (CBP), and general control non-depressible 5 (GCN5)] or co-repressors (c-Ski and SnoN) in the nucleus, leading to regulation of the transcription of target genes, including inhibitor of DNA binding (*ID*)*1*. Inhibitory Smads (I-Smads; Smad6, and Smad7) repress TGF-β family signaling, mainly through interaction with type I receptors (Miyazawa & Miyazono, 2017). While both TGF-β signaling and BMP signaling are inhibited by Smad7, Smad6 preferentially inhibits BMP signaling through ALK3 and ALK6 (Goto et al., 2007). In addition to these Smad-dependent signaling pathways, BMPs activate non-Smad signaling pathways, including the extracellular signal-regulated kinase (Erk), c-Jun N-terminal kinase (Jnk), p38 mitogen-activated protein (MAP) kinase, phosphoinositide 3 (PI3) kinase-Akt, and small GTPase pathways. Non-Smad signaling pathways cooperate with Smad signaling pathways to regulate various cellular responses.

## Altered Expression of Bone Morphogenetic Proteins and Their Signaling Components in Cancers

Altered expression of BMPs or related signaling components has been observed in many cancers. In addition to conventional histopathological analyses using clinical specimens, analyses of public databases have indicated the clinical significance of BMPs in cancer progression. Here, we introduce recent findings for each type of cancer.

### Hepatocellular Carcinoma

Numerous reports have suggested that BMP signaling is activated in hepatocellular carcinoma (HCC) tissues. The expression levels of BMP-2/4 and ALK3 were upregulated in tumor tissues. Many of these are correlated with various clinical or biological parameters, such as unfavorable prognosis, tumor grade, TNM stage, vascular invasion, and the expression of the stem cell marker CD133 (Guo D et al., 2012; Zhang et al., 2012; Ma et al., 2017; Feng et al., 2019; Li et al., 2020; Guo et al., 2021). Increased BMP-9 expression is also observed in HCCs and significantly associated with poor outcomes and the T stage of HCC (Herrera et al., 2013; Li et al., 2013; Chen et al., 2021). Of note, high levels of BMP-9 were detected, especially at the tumor borders, in samples from an HCC mouse model (Li et al., 2013). Although conflicting data have also been reported (Wang et al., 2018), the pro-oncogenic function of BMP signaling in HCC has been supported by many reports.

### Colorectal Cancer

It is well known that germline mutations in *MADH4* or *BMPR1A* (encoding Smad4 or ALK3, respectively) are present in a large number of patients with juvenile polyposis syndrome (Ehata et al., 2013). Phosphorylation of Smad1/5 has been reported to be absent in most colorectal cancers (CRCs). A recent study estimated that methylation of *BMP2* occurs in 60.2% of sporadic CRCs (Miura et al., 2020). Based on these findings, many reports have indicated that BMPs act as tumor suppressors in CRC. On the contrary, BMP signaling also acts as a pro-tumorigenic factor by promoting tumor invasion, epithelial-mesenchymal transition (EMT), and tumor proliferation (Davis et al., 2016). Fan et al. revealed that BMP-9 expression gradually increased during the transition from normal mucosa to adenoma and subsequent adenocarcinoma in the colon (Fan et al., 2020). Our analysis showed that BMP-4 was highly expressed in CRC tissues compared to normal tissues, and that BMP-4 is involved in CRC progression as an autocrine factor (Yokoyama et al., 2017). One possible explanation for the different roles of BMPs in CRC might be the BMP-induced non-Smad signaling pathway. Based on the expression of the BMP receptor and Smad4, Voorneveld. et al. subdivided CRC cases and found that the expression of normal BMP receptors in Smad4-negative tumors was associated with poor prognosis, suggesting that Smad4-independent BMP signaling may accelerate the progression of CRC (Voorneveld et al., 2014). They also reported that patients with a combination of high BMP-2 expression in the stroma and loss of Smad4 in tumors showed a significantly poorer overall survival (Ouahoud et al., 2020).

### Lung Cancer

In non-small cell lung cancers, elevated serum BMP-2 levels are observed in many patients, and they serve as a marker for effective treatment (Choi et al., 2012; Fei et al., 2013). In contrast, Liu et al. revealed that in small cell lung cancers, BMP-7 expression was not detected in cancer tissues, and that BMP-7-positive tumors were correlated to the absence of bone metastasis (Liu et al., 2012).

### Breast Cancer

There are still conflicting data regarding the role of BMPs in the progression of breast cancer. The activation of Smad-dependent BMP signaling has been observed in primary and metastatic bone tumors in breast cancer (Katsuno et al., 2008; Owens et al., 2015). BMP-2/5/6/7 are observed in breast cancers and are correlated with the expression of the stem cell marker CD44 (Owens et al., 2015; Huang et al., 2017). The expression of *BMPR1A* (encoding ALK3) is correlated with poor relapse-free survival (Pickup et al., 2015). However, an inverse correlation was observed. BMP-5 expression is decreased in invasive breast cancer, and is associated with cancer recurrence (Romagnoli et al., 2012). The expression of BMP-9 is significantly decreased in the breast cancer tissues, compared with paracancerous tissues (Li et al., 2018). The expression of BMP antagonists, such as noggin or follistatin, is correlated to bone metastasis, but not to metastasis to other organs (Tarragona et al., 2012; Mock et al., 2015).

### Renal Cell Carcinoma

Although BMP-9 expression is found over 80% of RCC (Wang et al., 2016), recent studies mainly support the tumor-suppressive role of BMPs in renal cell carcinoma (RCC). BMP-2 is downregulated by the promoter CpG methylation of *BMP2* in RCC cells. The resultant loss of BMP-2 is correlated to poor prognosis in RCC (Mitsui et al., 2015). Consistent with these findings, we observed increased expression of the transcriptional co-repressor c-Ski in cancer cells in RCC tissues (Taguchi et al., 2019). However, it should be mentioned that c-Ski may accelerate cancer progression mainly through the suppression of TGF-β-dependent Smad signaling, and not through the suppression of BMP-dependent Smad signaling in an experimental setting. Our latest data also indicated that endoglin expression was heterogeneously upregulated in highly malignant derivatives obtained after serial transplantation of human RCC cells (Momoi et al., 2021).

### Ovarian Cancer

The expressions of BMPs and their signaling components, BMP-2/7, ALK2/3, BMPR-II, and phosphorylated Smad5, are elevated in ovarian cancers (Hover et al., 2015; Peng et al., 2016; Guan et al., 2019; Fukuda et al., 2020). These findings consistently suggest pro-oncogenic roles of BMPs. Moreover, Fukuda et al. observed high BMP-2 expression after chemotherapy for ovarian cancer (Fukuda et al., 2020). The role of anti-Müllerian hormone (AMH), a member of the TGF-β family, has also been investigated in ovarian cancers. AMH activates the Smad1/5-mediated signaling pathway through the binding to AMH type II receptors (AMHRII) and type I receptors (ALK2/3/6). AMH is produced by granulosa cells in females, and it acts as a key factor in sexual differentiation. Interestingly, AMH has been shown to inhibit the proliferation of ovarian granulosa cell tumor cells (Anttonen et al., 2011) and epithelial ovarian cancer cells (Zhang et al., 2018). Further analyses are needed to elucidate the role of BMP signaling in ovarian cancer.

### Endometrial Cancer

High expression of *BMP2* and *BMP7* mRNA is associated with poorer survival in patients with endometrial cancer, albeit not significantly for *BMP2* (Fukuda et al., 2021). In addition, mutations in *ACVR1,* the gene encoding ALK2, are more frequently observed in endometrial cancer (>6%) than in most other cancers. Mutations in *ACVR1* were originally found in patients with fibrodysplasia ossificans progressiva (FOP) (Shore et al., 2006), and have also been found in pediatric brain tumors (see below). The mutant ALK2 in the GS domain (R206H) shows hyperactivation of the kinase domain, and it acquires the ability to bind activins and BMPs (Hatsell et al., 2015). Concerning the expression of BMP-10, a controversial implication has also been reported in endometrial cancer (Hu et al., 2021).

### Prostate Cancer

In prostate cancer, decreased BMP-2 expression in cancer tissue is correlated to recurrence and the Gleason score, which represents histological patterns and prognosis (Tae et al., 2018). We have also reported that BMP-7 inhibits the proliferation of prostate cancer cells in cell culture and a xenograft model (Miyazaki et al., 2004). However, BMP-7 expression in metastatic prostate cancer tissues is associated with shorter patient survival, suggesting a context-dependent contribution of BMPs in prostate cancer (Liu et al., 2015).

### Squamous Cell Carcinoma in the Esophagus, Head, and Neck

Two independent groups revealed that high BMP-7 was observed in primary esophageal cancer tissues, with a correlation to prognosis and metastasis-related parameters (Megumi et al., 2012; Xu et al., 2013). Interestingly, the level of phosphorylated Smad1/5 is elevated in the tumor tissues of patients with cetuximab-resistant oral squamous cell carcinoma with poor prognosis (Yin et al., 2018).

### Nasopharyngeal Carcinoma

High BMP2 expression is significantly associated with clinical stage, distant metastasis, and shorter survival in patients with nasopharyngeal carcinoma (Wang et al., 2017).

### Glioma

BMPs suppress the tumorigenic function of human glioma-initiating cells by inducing cell differentiation, cell cycle arrest, and apoptosis (see below). Accordingly, several reports have shown that BMP-4 is expressed in low-grade gliomas, and that it serves as a favorable prognostic marker in gliomas (Bao et al., 2013; Nayak et al., 2020). BMP-4 is overexpressed in gliomas harboring *IDH1* mutations, which are a hallmark of better prognosis (Zhou et al., 2020).

In contrast, in diffuse intrinsic pontine glioma (DIPG), a glial tumor in the brainstem with highly infiltrative properties in children, mutations in *ACVR1* may exhibit pro-oncogenic functions. Four independent research groups reported that *ACVR1* mutations were found in approximately 20–30% of DIPG (Buczkowicz et al., 2014; Fontebasso et al., 2014; Taylor et al., 2014; Wu et al., 2014). Notably, many somatic *ACVR1* mutations in DIPG are identical to germline mutations found in FOP. However, given that FOP is not accompanied by a tumor predisposition, other oncogenic mechanism(s) are required for the pathogenesis of DIPG. The increased activation of *ACVR1* may contribute to the pathogenesis of DIPG in the presence of several histone modifications. Co-expression of *ACVR1* G328 and histone H3.3K27M additively increased the expression of *ID1* and *ID2* (Buczkowicz et al., 2014). Evolutionary analysis showed specific associations between H3K27M and mutations in *TP53*, *PPM1D*, *ACVR1*, and *PIK3R1* (Nikbakht et al., 2016). Hoeman et al. recently demonstrated that *ACVR1* R206H or G328V with H3.1K27M activates signal transducer and activator of transcription 3 (STAT3) signaling (Hoeman et al., 2019). Inhibition of BMP signaling may suppress the progression of *ACVR1* mutation-positive DIPG (Carvalho et al., 2019). However, how BMP signaling affects the progression of *ACVR1* mutation-negative DIPG cells remains unknown. It should also be noted that mutations in the *ACVR1* gene are observed in other types of cancers, including endometrial cancer, although the frequencies of the *ACVR1* mutations are lower than those in DIPG (Fukuda et al., 2021).

## Effect of Bone Morphogenetic Proteins on the Proliferation and Survival of Cancer Cells

**FIGURE 2:**
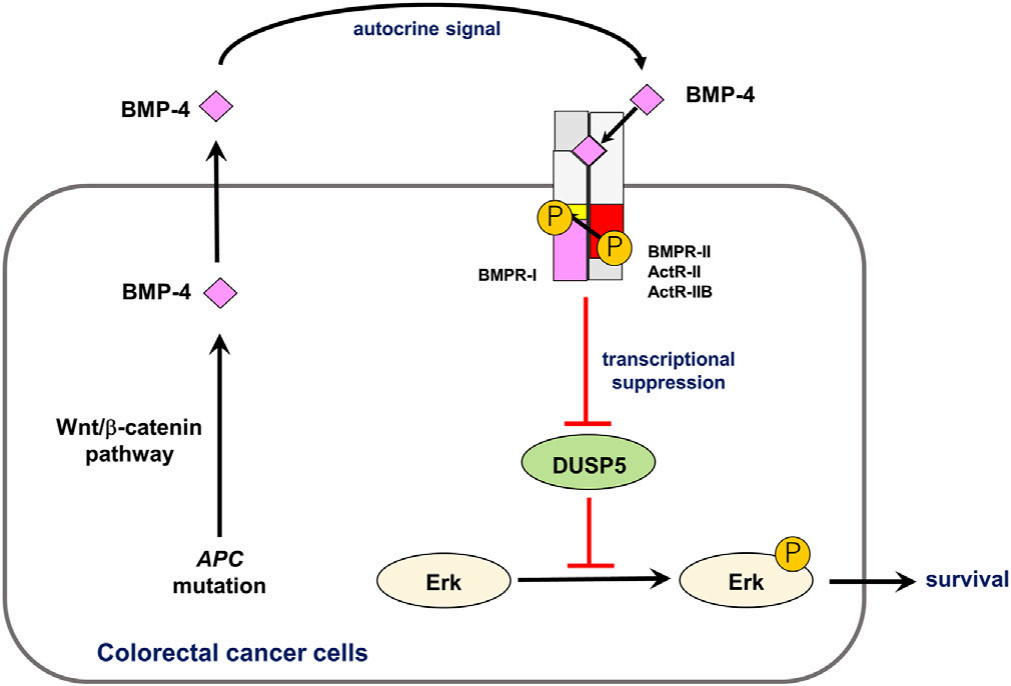
Role of autocrine BMP-4 signaling in CRC. Aberrant activation of the Wnt/β-catenin pathway induces expression of *BMP4* mRNA, activating endogenous BMP signaling. This endogenous signaling promotes phosphorylation of Erk MAP kinase *via* DUSP5 suppression, which results in survival of CRC cells. Modified from Yokoyama et al. (2017).

BMPs promote the progression of many types of cancers through the activation of proliferation and survival of cancer cells (Ehata et al., 2013). The detailed mechanisms of these pro-oncogenic roles of BMPs have recently been uncovered, especially in HCC. BMP-4 upregulates the expression of cyclin-dependent kinase (CDK) 1 and cyclin B1 in HCC cells and accelerates cell cycle progression by activating Erk MAP kinase (Chiu et al., 2012). Ma et al. reported that exogenous BMP-4 increases the expression of cyclin A and CDK2 and promotes the G1-S phase transition in several HCC cells. The BMP-4-mediated cell proliferation was attenuated by silencing ID2 (Ma et al., 2017). The human HCC cell line HepG2 produces BMP-9 in an autocrine fashion, which triggers cell cycle progression and abolishes apoptosis induced by serum starvation. Notably, BMP-9 promotes the growth of HCC cells, but not of immortalized human hepatocytes, suggesting that other oncogenic signaling pathways may modulate the effect of BMP-9 (Herrera et al., 2013). BMP-4 has also been shown to promote the proliferation of HCC cells *via* autophagy activation through Jnk1/Bcl-2 signaling (Deng et al., 2018). In addition to HCC, the pro-survival effect of BMP signaling is indicated in other cancers. We have revealed that aberrant activation of the Wnt/β-catenin pathway induces *BMP4* mRNA expression, activating endogenous BMP signaling in CRC cells (Figure 2). This endogenous signaling enhances the phosphorylation of Erk MAP kinase through the downregulation of dual-specificity phosphatase 5 (*DUSP5*), thereby promoting the survival of CRC cells (Yokoyama et al., 2017). Stress-induced phosphoprotein 1 (STIP1) is secreted by ovarian cancer cells. The binding of STIP1 to ALK2 activates the Smad signaling pathway, leading to the transcriptional activation of ID3, promoting cell proliferation (Tsai et al., 2012). Most of *ACVR1* mutations in DIPG cause constitutive activation of ALK2, which increases the expression of the downstream targets *ID1*, *ID2*, and *SNAI1* (encoding Snail), and also increases cell proliferation (Buczkowicz et al., 2014; Fontebasso et al., 2014). Treatment of tumor cells with a small-molecule ALK2 inhibitor, LDN-193189, attenuated their viability (Taylor et al., 2014).

In contrast, BMPs have been shown to negatively regulate cell cycle progression in other types of cancer cells, including gastric and prostate cancer cells (Ehata et al., 2013). We previously found that BMP-4 induces G1 arrest in diffuse-type gastric carcinoma cells *via* induction of p21 through the Smad pathway, inhibiting cell proliferation (Shirai et al., 2011). Similarly, BMP-2 inhibits esophageal cancer cell growth by inducing p21 through the Smad pathway and activating the Hippo signaling pathway (Kim et al., 2016). BMP-7 also inhibits prostate cancer cell proliferation by inducing p21 and suppressing CDK2 activity (Miyazaki et al., 2004). BMP-10 suppresses the proliferation of HCC cells by inhibiting the signal transducer and activator of transcription 3 (STAT3) signaling. Mechanistically, cytoplasmic BMP-10 interacts with both protein tyrosine phosphatase sigma (PTPRS) and STAT3, thus facilitating the dephosphorylation of STAT3 by PTPRS (Yuan et al., 2019). Olsen et al. reported that BMP-9 induces apoptosis in multiple myeloma cells through the ALK2-mediated signaling pathway (Olsen et al., 2014). As different types of cancers cannot account for the diverse effects of BMPs, they are thought to regulate the proliferation or survival of each cancer cell in a context-dependent manner.

## Effect of Bone Morphogenetic Proteins on Cancer Stem Cells

**FIGURE 3:**
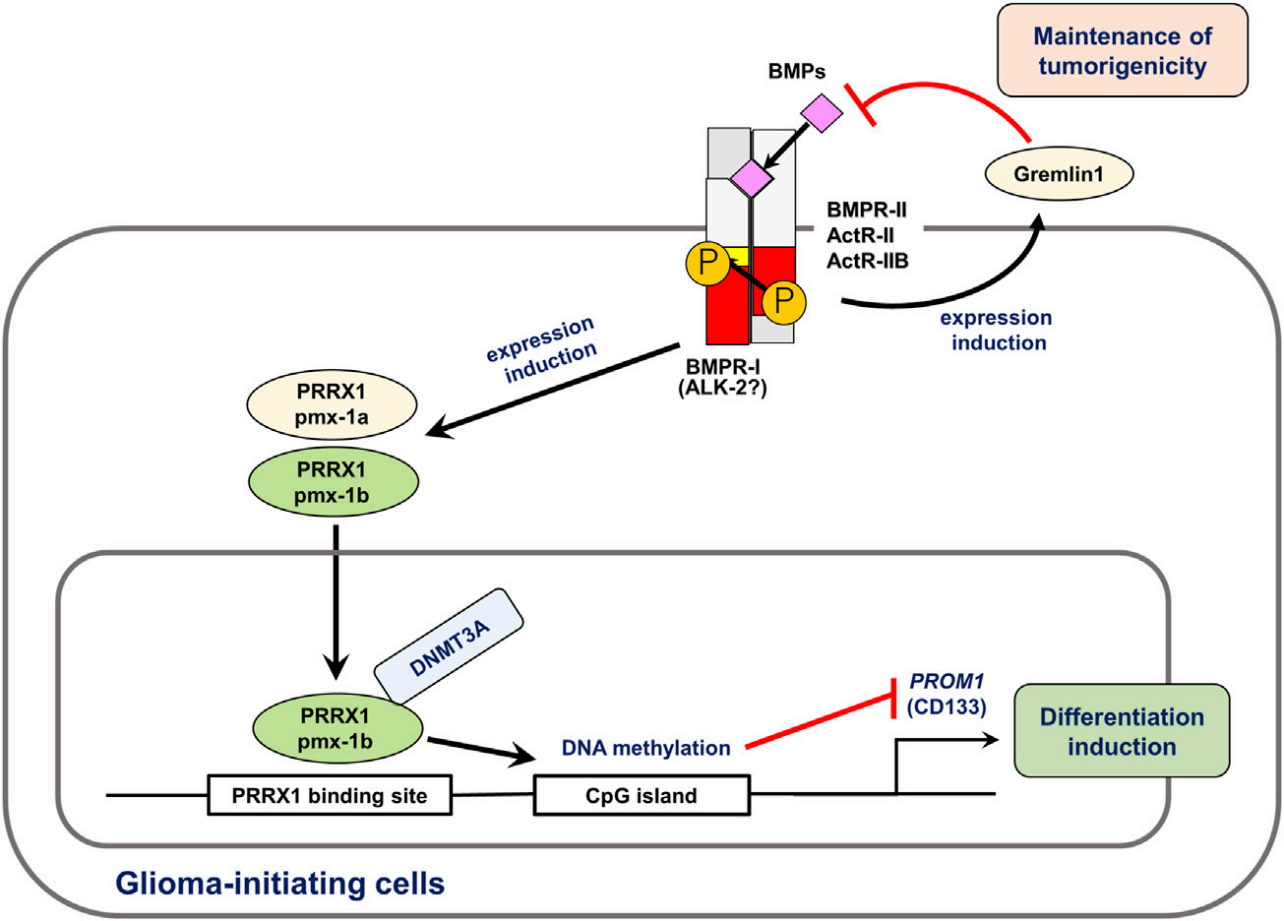
BMPs induce differentiation and apoptosis of glioma-initiating cells. BMPs regulate expression of various target genes in glioma-initiating cells. In this figure, the roles of PRRX1 (Tanabe et al., 2022) and gremlin1 (Yan et al., 2014) are shown. Of the two splice isoforms of PRRX1, only pmx-1b induces differentiation of glioma-initiating cells. Through interaction with DNMT3A, pmx-1b induces the methylation of the *PROM1* gene promoter and suppressed the CD133^+^ glioma-initiating cell population. BMPs induce the expression of gremlin1, an antagonist of BMP, which is involved in the maintenance of pro-tumorigenic functions and stem cell properties of glioma-initiating cells. Modified from Tanabe et al. (2022).

Cancer stem cells (CSCs) or cancer-initiating cells may be responsible for cancer recurrence. As BMPs act as differentiation factors in several organs, the activity of CSCs is diminished by BMPs. BMPs induce differentiation of glioma-initiating cells, leading to cell cycle arrest or apoptosis (Piccirillo et al., 2006; Lee et al., 2008). Various mechanisms have been determined for the BMP-mediated differentiation of glioma-initiating cells. BMPs induce the EMT-associated transcription factor Snail *via* Smad-dependent pathways, which results in the deletion of tumorigenic potential (Savary et al., 2013). As a mechanism, Snail transcriptionally represses *TGFB1* through interaction with Smads, thereby regulating astrocytic differentiation (Caja et al., 2018). Our research group has also shown that distal-less homeobox 2 (DLX2), an essential transcription factor induced by BMPs, is important for neural differentiation and apoptosis of glioma-initiating cells (Raja et al., 2017). In addition to these transcription factors, BMPs affect glioma-initiating cells *via* epigenetic mechanisms. We found that the expression of paired related homeobox 1 (PRRX1) is induced by BMPs in glioma-initiating cells. The longer isoform of PRRX1, pmx-1b, interacts with DNA methyltransferase 3A (DNMT3A) and induces promoter methylation of the *PROM1* gene encoding CD133, thereby attenuating stem cell-like properties (Tanabe et al., 2022) (Figure 3). We have also reported that the tyrosine kinase Eph receptor A6 (EPHA6) promotes apoptosis in BMP-2-sensitive glioma-initiating cells (Raja et al., 2019). BMPs thus play tumor-suppressive roles in the progression of glioma, acting on glioma-initiating cells. However, some glioma cells acquire resistance to the action of BMP-Smad signaling pathways, and several extracellular proteins are associated with the sensitivity of some glioma cells to BMPs. Increased expression of the extracellular antagonists gremlin1 or follistatin-like 1 promotes the maintenance of glioma-initiating cells through the attenuation of BMP signaling (Yan et al., 2014; Jin et al., 2017).

Likewise, the effect of BMPs on the differentiation of CSCs has been investigated in cancers other than glioma. Although TGF-β-induced EMT may render breast cancer cells with stem cell properties, BMPs, particularly heterodimeric BMP-2/7, antagonize TGF-β-induced EMT and reduce the aldehyde dehydrogenase (ALDH)^h^ CD44^h^ CD24^l−^ CSC population (Buijs et al., 2012). In normal mammary epithelial cells, BMP-4 acts as a pro-differentiation factor and promotes acinar formation. However, in triple-negative breast cancer (TNBC) cells, TGF-β inhibits the expression of BMP-4 through the Smad pathway and cyclin D1. Thus, TGF-β enhances tumor formation and increases the highly tumorigenic CD44^high^ CD24^low^ CSC population (Yan et al., 2021). Whissel et al. found that *BMP4* transcription is regulated by GATA-binding protein 6 (GATA6), which is related to the self-renewal of adenoma stem cells in the colon (Whissell et al., 2014). In RCC cell lines, BMP-2 downregulates the expression of stem cell markers in ALDH^+^ cells and potently inhibits their growth (Wang et al., 2015). CSC-like cells in head and neck cancer display decreased levels of phosphorylated Smad1/5 and BMP target gene *ID1*, whereas Smurf1, a negative regulator of BMP signaling, is highly expressed compared to that in non-CSC populations. When BMP signaling pathways are reactivated by Smurf1 knockdown in CSC-like cells, adipogenic differentiation and loss of tumorigenic capacity are observed (Khammanivong et al., 2014). BMP-2 is thought to act as a potent inducer of tumor cell transdifferentiation in osteosarcoma (Geng et al., 2014).

These observations indicate that BMPs promote the differentiation of CSCs and attenuate the tumor-forming capability of several cancer cells. Thus, BMP administration is thought to be suitable for these cancers (Reguera-Nuñez et al., 2014; Nayak et al., 2020). For example, exogenous BMP-4 induces the differentiation of CSCs within HCC through the activation of the Erk1/2 MAP kinase signaling pathway, inhibiting the self-renewal and tumorigenic capacities of CD133^+^ CSCs. Simultaneously, the expression of one of the ABC transporters, ATP-binding cassette subfamily G member 2 (ABCG2), is decreased in hepatic cancer stem cells after BMP-4 treatment, which enhances sensitivity to chemotherapeutic agents (Zhang et al., 2012). González-Gómez et al. employed BMP-7-loaded microspheres in a xenograft model and demonstrated that controlled release of BMP-7 potently inhibited the growth of glioblastoma and reduced CSC markers, including CD133, Olig2, and glial fibrillary acidic protein (GFAP)δ (González-Gómez et al., 2015).

However, BMPs are also important for the maintenance of CSCs in HCC. Zhang et al. revealed that high-dose exogenous BMP-4 promotes the differentiation of CD133^+^ CSCs in HCC, whereas low-dose exogenous BMP-4 upregulates CD133 expression, suggesting a concentration-dependent effect of BMPs on CSC maintenance (Zhang et al., 2012). Silencing of BMP-2 in HCC suppresses Erk MAP kinase and inhibits sphere formation and the expression of stemness-related and EMT markers in CSCs from HCC cell lines (Guo et al., 2021). BMP-9 promotes CSC properties in epithelial cell adhesion molecule (EpCAM)-positive HCC subtypes by enhancing ID1 expression (Chen et al., 2021).

## Effect of Bone Morphogenetic Proteins on Cancer Cell Migration, Invasion, and Metastasis

Similar to TGF-β, BMPs enhance the migration and invasion of several types of cancer cells, thereby potentiating their metastatic ability. This pro-metastatic function of BMPs has been supported by numerous recent studies (Guo X et al., 2012; Maegdefrau & Bosserhoff, 2012; Xu et al., 2013; Leinhäuser et al., 2015; Liu et al., 2015; Guan et al., 2019; Huang et al., 2020). The BMP-Smad pathway plays a critical role in the induction of EMT in many cancer cells. For example, BMP-4 induces the expression of the EMT-associated transcription factors *SNAI1* and *SNAI2*, which encode Snail and Slug, respectively, in a Smad4-dependent manner in ovarian and pancreatic cancer cells. This regulatory mechanism is critically dependent on matrix rigidity and Yes-associated protein 1 (YAP1) (Serrao et al., 2018). BMP-4 enhances EMT and stem cell properties *via* the Smad-dependent pathway in both mammary epithelial and breast cancer cells, which is accompanied by the activation of Notch signaling (Choi et al., 2019). In HCC cell lines, autocrine BMP-9 signaling induces Snail expression *via* the ALK1- and ALK2-Smad1 pathways (Li et al., 2013).

Non-Smad pathways, including PI3 kinase-Akt, mTOR, and Rock, are involved in BMP-mediated EMT. BMP-2 induces EMT and stemness of breast cancer cells through the Rb and CD44 signaling pathways, which act not only *via* Smad-dependent pathways but also *via* the PI3 kinase-Akt signaling pathway (Huang et al., 2017). The mTORC1 inhibitor rapamycin blocks BMP-2-induced EMT in nasopharyngeal carcinoma cells (Wang et al., 2017). In CRC, Smad4-independent BMP signaling induces EMT and invasion *via* the Rock pathway (Voorneveld et al., 2014). In chondrosarcoma cells, BMP signaling regulates the expression of matrix metalloproteinases (MMPs) and promotes invasiveness *via* non-Smad signaling pathways, including p38 MAP kinase and Akt pathways (Chen et al., 2014; Yahiro et al., 2019).

Despite these findings, exogenous BMP-2 does not affect the metastatic phenotype of osteosarcoma cells (Gill et al., 2017). Moreover, the suppressive effects of BMPs on metastasis have been documented in breast cancer and osteosarcoma (Ren et al., 2014a; Ren et al., 2014b; Xiong et al., 2018). Overexpression of some BMP antagonists, such as gremlin1 or noggin, promotes metastasis of breast cancer cells (Tarragona et al., 2012; Sung et al., 2020). Although the reason for these conflicting effects remains unclear, the experimental methods employed in each report might have affected the function of BMPs.

## Effect of Bone Morphogenetic Proteins on Tumor Angiogenesis

ALK1 is predominantly expressed in proliferating vascular endothelial cells. As BMP-9/10 have been identified as ligands for ALK1, the role of BMP-9/10-ALK1 signaling in vascular angiogenesis has been well established (see Figure 1) (Ehata et al., 2013). The BMP-9/10-ALK-1 signaling pathway activates the proliferation of endothelial cells under certain conditions, and plays an important role in the maintenance of vascular homeostasis. We previously showed that BMP-9 induces tumor angiogenesis in a mouse xenograft model of human pancreatic cancer (Suzuki et al., 2010). However, the effect of BMP-9/10-ALK1 signaling on lymphatic endothelial cells may differ from that on vascular endothelial cells. Yoshimatsu et al. revealed that BMP-9 directly downregulates prospero homeobox 1 (Prox1) expression *via* ALK1 in human dermal lymphatic endothelial cells (HDLECs) and reduces their proliferation. BMP-9 was shown to inhibit tumor lymphangiogenesis in a mouse breast cancer allograft model (Yoshimatsu et al., 2013).

In contrast to BMP-9/10-ALK1 signaling, the role of ALK2/3/6-mediated BMP signaling in angiogenesis remains controversial. Elevated expression of BMP-2 is found in HCC and is positively correlated to angiogenesis in tumor tissues. A loss-of-function assay using shRNAs revealed that BMP-2 secreted from HCC cells activates the p38 MAP kinase signaling pathway in endothelial cells, thus enhancing the proliferation, migration, and angiogenic abilities of endothelial cells (Feng et al., 2019). BMP-7 exhibits a pro-angiogenic effect through the expression of repulsive guidance molecule family member B (RGMb), a co-receptor for BMPs, in endothelial cells (Sanders et al., 2014). Taken together, the impairment of tumor angiogenesis may be achieved by blocking BMP signaling pathways other than BMP-9/10-ALK1 (Jia et al., 2016). However, under certain conditions, the direct administration of BMPs to tumor-bearing mice exerts an anti-angiogenic effect. Intraperitoneal treatment with BMP-4 suppressed tumor angiogenesis and reduced tumor formation in xenograft and allograft models of some cancer cells. As its mechanism, BMP-4 reduces vascular endothelial growth factor (VEGF) expression *in vivo* in a thrombospondin 1 (TSP1)-dependent manner (Tsuchida et al., 2014). BMP-7v, a modified BMP-7, is capable of reducing the number of microvessels in CSC-based avatars, resulting in the sensitization of CRC cells to chemotherapeutic reagents (Veschi et al., 2020).

## Effect of Bone Morphogenetic Proteins on Other Cellular Components in the Tumor Microenvironment

BMPs mediate the interactions between cancer cells and various cellular components in the tumor microenvironment. BMP-4 expression is upregulated in cancer-associated fibroblasts (CAFs) in HCC tissues compared to non-cancerous liver fibroblasts. BMP-4 overexpression in normal fibroblasts activates these cells to a CAF-like phenotype. Simultaneously, BMP-4 enhances the production of interleukin (IL)-6, IL-8, and chemokine (C-C motif) ligand (CCL)2 and promotes cancer cell invasion (Mano et al., 2019). BMP-2 is upregulated in fibroblasts upon stimulation with conditioned medium from Smad4-deficient CRC cells, which in turn increases the liver metastasis of Smad4-deficient CRC cells, but not that of Smad4-proficient CRC cells. BMP-2 expression in fibroblasts appears to be regulated by tumor necrosis factor (TNF)-related apoptosis-inducing ligand (TRAIL) derived from Smad4-deficient CRC cells. Thus, a reciprocal loop in which TRAIL from Smad4-deficient CRC cells induces BMP-2 in fibroblasts plays a critical role in cancer progression (Ouahoud et al., 2020). BMPs and hedgehogs mediate the interactions between cancer cells and the tumor microenvironment. We previously reported that BMP-4 induces the production of sonic hedgehog in prostate cancer cells, thereby enhancing osteoblastic differentiation of stromal cells and may account for osteoblastic metastasis of prostate cancer (Nishimori et al., 2012). Hedgehogs secreted from ovarian tumor cells induce BMP-4 expression in carcinoma-associated mesenchymal stem cells. BMP-4 reciprocally increases hedgehog expression in ovarian tumor cells, indicating a positive feedback loop. The interruption of this loop with a hedgehog pathway inhibitor or BMP-4-blocking antibody prevents the enrichment of CSCs and reverses chemotherapy resistance (Coffman et al., 2016).

BMPs suppress anti-tumor immunity (reviewed in Sconocchia & Sconocchia, 2021). Recent studies revealed that BMP signaling is shown to be involved in the activation of macrophages or dendritic cells during cancer progression. Bladder cancer cells produce BMP-4, which enhances the macrophage polarization toward anti-inflammatory M2 phenotype (Martinez et al., 2017). BMP-4 derived from acute lymphoblastic leukemia cells promotes the generation of dendritic cells with immunosuppressive features and polarizes macrophages towards a less pro-inflammatory phenotype (Valencia et al., 2019). Ihle et al. utilized a LysMCre-mediated myeloid-specific *Bmpr1a* conditional knockout mouse model along with a syngeneic prostate cancer model and demonstrated the pro-tumorigenic role of ALK3 in myeloid cells. They also confirmed that macrophage polarization is altered by ALK3 inhibition in this setting (Ihle et al., 2020). BMP-7 is upregulated in tumors in a mouse model that does not respond to treatment with immune checkpoint inhibitors (Cortez et al., 2020). Mechanistically, tumor cell-derived BMP-7 downregulates MAP kinase (MAPK) 14, which regulates some cytokines and chemokines, including *IL1A*, *IL1B*, *TNF*, and *CCL5 via* Smad1 activation in macrophages.

The role of BMPs on the lymphocyte functions has recently been investigated. Kuczma et al. extracted *Bmpr1a* as a gene that regulate the immune suppressive function of regulatory T cells (Treg) (Kuczma et al., 2014). Inactivation of *Bmpr1a* in T cells resulted in impaired generation of Treg, leading to the reduced tumor growth in mice bearing B16 melanoma cells. BMP-7 decreases CD4^+^ T-cell activation by downregulating interferon γ and IL-2 expression *via* Smad/MAPK14 signaling, resulting in resistance to immunotherapy (Cortez et al., 2020). These findings support the idea that inhibition of BMP signaling may be beneficial for cancer treatment.

However, other reports have shown that BMPs suppress the function of tumor-promoting immune cells (Sconocchia & Sconocchia, 2021). BMP-4 reduces the secretion of granulocyte colony-stimulating factor (G-CSF) from mammary tumors, which is likely a critical factor for the expansion of myeloid-derived suppressor cells (MDSCs) and progression of metastasis (Cao et al., 2014). Although the anti-inflammatory effects of TGF-β are well established, the precise role of BMP signaling in the immune system remains fully uncovered.

## Applications of Small-Molecule Bone Morphogenetic Protein Inhibitors in Cancer Treatment

Many researchers have attempted to use BMP type I receptor inhibitors to treat various types of cancers. As mutations in the *ACVR1* gene are responsible for the pathogenesis of FOP, and such mutations are also found in patients with DIPG, the development of ALK2-specific inhibitors is expected. The current computational approach reveals small changes in the binding site residue type or side-chain orientation in ALKs, as well as subtle structural modifications of the inhibitors, which can be used to improve the specificity of BMP inhibitors (Alsamarah et al., 2015). Dorsomorphin is a prototype BMP type I receptor inhibitor containing a pyrazolo [1,5-a]-pyrimidine scaffold. Subsequently, more ALK2-selective and metabolically stable inhibitors, LDN-193189 and LDN-212854, were developed (Rooney & Jones, 2021). In addition to pyrazolo [1,5-a]-pyrimidine, other scaffolds have also been identified, such as pyridines, quinazolinones, and pyrazoles. M4K2163, a pyridine-based compound, has been shown to have good permeability in the brain (Rooney & Jones, 2021). Our group recently developed the pyrazole-based compounds RK-59638 and RK-71807 (Sato et al., 2020).

These bioavailable inhibitors have allowed examination of their beneficial effects in various types of mouse tumor models. The therapeutic effects of these inhibitors have been documented for DIPG. Treatment of mice bearing *ACVR1* R206H mutation-harboring DIPG cells with LDN-193189 or LDN-214117 extended the survival of host mice compared with vehicle controls (Carvalho et al., 2019). Many reports have also shown that ALK2 inhibitors potently inhibit the formation and progression of other cancers. In breast cancer, treatment of a mouse cancer model with LDN-193189 suppressed tumor-initiating capacity and EMT induction, as well as prolonged tumor latency (Balboni et al., 2013). When MMTV.PyVmT-expressing mice were treated with an osmotic pump containing the BMP type I receptor inhibitor, DMH1, the tumors were less proliferative and more apoptotic, reducing lung metastasis. Simultaneously, DMH1 affects fibroblasts, lymphatic vessels, and macrophages, reducing their tumor-promoting effects (Owens et al., 2015). Our group revealed that intraperitoneal administration of LDN-193189 reduced the Erk MAP kinase signaling pathway in CRC cells, attenuating primary tumor formation in mice-bearing CRC cells (Yokoyama et al., 2017). In HCC, treatment with LDN-212854 repressed ID1 and EpCAM expression in cancer cells *in vivo*, suggesting that the repression of the BMP-9-induced CSC phenotype was attenuated by the inhibitor (Chen et al., 2021). In brain tumors, ovarian cancer, lung cancer, oral squamous cell carcinoma, endometrial cancer, and melanoma, the therapeutic benefits of ALK2 inhibitors are suggested (Langenfeld et al., 2013; Hao et al., 2014; Hover et al., 2015; Peng et al., 2016; Newman et al., 2018; Yin et al., 2018; Mihajlović et al., 2019; Zhou et al., 2020; Fukuda et al., 2021; Kalal et al., 2021). Our research group recently reported that a BMP type I receptor inhibitor, RK-783, inhibited the growth of ovarian cancer cells *in vivo* (Fukuda et al., 2020).

As the survival of cancer cells is augmented by BMP signaling, BMPs determine the sensitivity to chemotherapeutic drugs (Camara-Clayette et al., 2013; Bach et al., 2018). ALK2 inhibitors are expected to be used in combination with cytotoxic agents to treat cancers. DMH1 enhances the sensitivity of ovarian cancer cells to cisplatin treatment (Hover et al., 2015). DMH1 reduces the growth of cetuximab-resistant oral squamous cell carcinoma (Yin et al., 2018). LDN-193189 augmented the growth-inhibitory effects of carboplatin (Fukuda et al., 2021).

However, these inhibitors are not entirely specific to ALK2. Among the pyrazolo [1,5-a]-pyrimidine-containing inhibitors, dorsomorphin typically induces autophagy in ovarian cancer cells, whereas LDN-193189 induces ROS-mediated apoptosis in the same cell lines. As this discrepancy is thought to be caused by the off-target effects of each inhibitor, the development of more specific ALK2 inhibitors is required (Ali et al., 2015). In addition, as mentioned above, because BMPs have tumor-suppressive functions under certain conditions, tumor formation or metastasis might be promoted through the inhibition of BMP signaling (Vollaire et al., 2019; Sharma et al., 2021). We should clarify which types of cancer ALK2 inhibitors are effective against.

## Targeting Angiogenesis *via* Inhibition of ALK1 Signaling

To suppress tumor angiogenesis, various strategies have been utilized to inhibit BMP-9/10-ALK1 signaling, such as soluble form receptors, neutralizing antibodies, and small-molecule receptor inhibitors. Dalantercept (ACE-041), a soluble form of ALK1, acts as a ligand trap for BMP-9/10, inhibiting the interactions between BMP-9/10 and ALK1. The therapeutic potential of dalantercept has been demonstrated in various mouse tumor models (Mitchell et al., 2010; Cunha et al., 2015; Hawinkel et al., 2016). Notably, dalantercept inhibits tumor angiogenesis by regulating signaling pathways other than VEGF signaling. Thus, dalantercept is considered a promising inhibitor for treating RCC, in which escape from VEGF-mediated tumor angiogenesis is critical (Philips & Atkins, 2014; Wang et al., 2016). Initial studies have shown that dalantercept is well tolerated in humans. However, clinical trials with patients with RCC have failed to show therapeutic benefits (Bendell et al., 2014; Voss et al., 2017; Voss et al., 2019). Clinical studies on patients with other cancers have revealed that dalantercept has insufficient efficacy in HCC, ovarian cancer, endometrial cancer, and head and neck cancer (Makker et al., 2015; Jimeno et al., 2016; Burger et al., 2018; Abou-Alfa et al., 2019). PF-03446962 is a fully humanized monoclonal antibody that targets and neutralizes human ALK1 (van Meeteren et al., 2012). Its pharmacokinetics and therapeutic benefits were evaluated in a preclinical model, followed by evaluations in humans (Hu-Lowe et al., 2011; Luu et al., 2012; Li et al., 2014). Although the results of a phase I study of PF-0334962 in patients with cancer initially supported further evaluation (Doi et al., 2016; Goff et al., 2016; Simonelli et al., 2016), phase II studies have demonstrated insufficient efficacy or unacceptable toxicity in the treatment of CRC, urothelial cancer, and mesothelioma (Necchi et al., 2014; Wheatley et al., 2016; Clarke et al., 2019). The small-molecule kinase inhibitor K02288 inhibits BMP-9-induced phosphorylation of Smad1/5 in human umbilical vein endothelial cells to reduce both Smad- and Notch-dependent transcriptional responses. K02288 caused dysfunctional vessel formation in a chick chorioallantoic membrane angiogenesis assay (Kerr et al., 2015). It is currently unknown why the inhibition of ALK-1 signaling failed to show clinical effects, and further studies on the *in vivo* effects of ALK-1 inhibitors on tumor angiogenesis are needed. Additional information will be forthcoming.

## Conclusion

In the present article, the diverse effects of BMPs are reviewed based on the latest literatures. As BMPs are considered potentially important therapeutic targets in some cancers, treatments with BMP inhibitors have been attempted. However, favorable results have not always been obtained. BMPs act in a context-dependent manner and become tumor suppressors under certain conditions. It is difficult to understand the fact that conflicting data have been obtained for the same cancers, even in the analyses using same cancer cell lines. The key factor(s) that switch the pro-oncogenic or tumor-suppressive functions of BMPs remains unknown. Different BMPs may act differently on each receptor. In addition, BMPs may act not only on many cancer cells but also on cellular components in the tumor microenvironment, leading to different results in different experimental settings. Thus, discovery of biomarkers which can discriminate the responses of certain cancers to BMPs is required.

Finally, the amounts of different BMPs biologically available in the cancer microenvironment should be considered. In the past, to observe cellular responses, many cancer cells were stimulated with ligands or overexpressed with BMPs. Although these evaluations have greatly contributed to our understanding of the diverse effects of BMPs, they do not always reproduce the roles of BMPs under physiological conditions. Notably, some biological effects of BMPs occur in a concentration-dependent or biphasic manner. In addition, crosstalk between BMPs and other signaling pathways may be important. In particular, crosstalk between BMP signaling and TGF-β signaling, which is a strong determinant of the metastatic phenotype of cancer cells, may be important. BMPs often exert their biological functions by enhancing or counteracting the effects of TGF-β. Therefore, evaluating not only the intensity of BMP signaling but also the intensity of TGF-β signaling may be needed.
